# Multi-walled carbon nanotubes complement the anti-tumoral effect of 5-Fluorouracil

**DOI:** 10.18632/oncotarget.26770

**Published:** 2019-03-12

**Authors:** Eloisa González-Lavado, Lourdes Valdivia, Almudena García-Castaño, Fernando González, Carmen Pesquera, Rafael Valiente, Mónica L. Fanarraga

**Affiliations:** ^1^ Grupo de Nanomedicina, IDIVAL-Universidad de Cantabria, 39011, Santander, Spain; ^2^ Dpto. Física Aplicada, Facultad de Ciencias, Universidad de Cantabria, 39011, Santander, Spain; ^3^ Unidad De Ensayos Clínicos, Oncología Médica y Medicina Paliativa, Hospital Valdecilla-IDIVAL 39011, Santander, Spain

**Keywords:** nanomaterial, nanocarrier, drug delivery, combined therapy, microtubule dynamics

## Abstract

Multiple-drug resistance in human cancer is a major problem. To circumvent this issue, clinicians combine several drugs. However, this strategy could backfire resulting in more toxic or ineffective treatments. Carbon nanotubes (CNTs), and particularly multi-walled nanotubes (MWCNTs), display intrinsic properties against cancer interfering with microtubule dynamics and triggering anti-proliferative, anti-migratory and cytotoxic effects *in vitro* that result in tumor growth inhibition *in vivo*. Remarkably, these effects are maintained in tumors resistant to traditional microtubule-binding chemotherapies such as Taxol^®^.

In the view of these properties, we investigate the use of MWCNTs in the development of *active-by-design* nanocarriers, attempting to enhance the effect of broadly-used chemotherapies. We compare the cytotoxic and the anti-tumoral effect of 5-Fluorouracil (5-FU) -an antimetabolite treatment of various forms of cancer- with that of the drug physisorbed onto MWCNTs. Our results demonstrate how the total effect of the drug 5-FU is remarkably improved (50% more effective) when delivered intratumorally coupled to MWCNTs both *in vitro* and *in vivo* in solid tumoral models. Our results demonstrate how using MWCNTs as anti-cancer drug delivery platforms is a promising approach to boost the efficacy of traditional chemotherapies, while considerably reducing the chances of resistance in cancer cells.

## INTRODUCTION

Most of the localized tumors are satisfactorily treated with surgery. Unfortunately, most aggressive cancers metastasize to distant organs and develop resistance to chemotherapy. For this reason, clinicians often need to combine different drugs to simultaneously interrupt cell proliferation pathways at various points, boosting the potential cytotoxic effect of the treatment. Regrettably, anti-cancer drug combination does not always work. For instances, Taxol^®^ (paclitaxel) and 5-Fluorouracil (5-FU) inhibit different and complementary mechanisms in cancer cell proliferation but, they have been reported to interfere with each other, questioning the clinical use of this drug combination parenterally [1]. As an alternative, drug co-encapsulation has also been suggested as a possible way to increase the therapeutic response of the Taxol^®^ + 5-FU mixture [2]. Here, we propose a new alternative to boost the chemotherapeutic effect of 5-FU with nanomaterials which intrinsically display antitumoral properties, interfering with cell proliferative mechanisms thus, complementing the cytotoxic effect of the drug.

Carbon nanotubes (CNTs) have shown excellent properties and applications in nanobiotechnology [3–5]. In particular, multi-walled CNTs (MWCNTs) can penetrate most biological barriers and infiltrate inside cells where they display unique biomimetic properties with the intracellular cytoskeletal polymers, mostly with microtubules [6]. These tubulin polymers, that are traditional targets for many anticancer therapies [7], share many properties with MWCNTs. Both self-assemble, have similar dimensions, are exceptionally resilient, and show comparable physical properties (for example, shear stress, bending stiffness and Young′s modulus) [6]. Their similarities prompt interaction *in vitro* [8] and *in vivo* [9], and the assemblage mixed functional bio-synthetic tubulin polymers that display an enhanced stability compared to conventional microtubules [10]. The increased stability of these mixed polymers causes critical changes in the cellular biomechanics, triggering the reported anti-proliferative [9, 11], anti-migratory [12–14] and cytotoxic effects *in vitro* in cancer cells [15–17], and significant anti-tumoral effects *in vivo* [18, 19]. In addition, some studies show how MWCNTs are effective in cells and tumors that have developed resistance to Taxol^®^ [18]. Thus, the microtubule-stabilizing effect of Taxol^®^ -that binds a structural pocket in the β-tubulin polypeptide- could be significantly boosted when combined with MWCNTs, for these nanomaterials utilize an alternative microtubule stabilization mechanism, complementary to that Taxol^®^ [10]. Summarizing, these results support the potential use of MWCNTs as anti-tumoral agents exclusively based on their intrinsic properties, using novel cytotoxic mechanisms.

**Figure 1:**
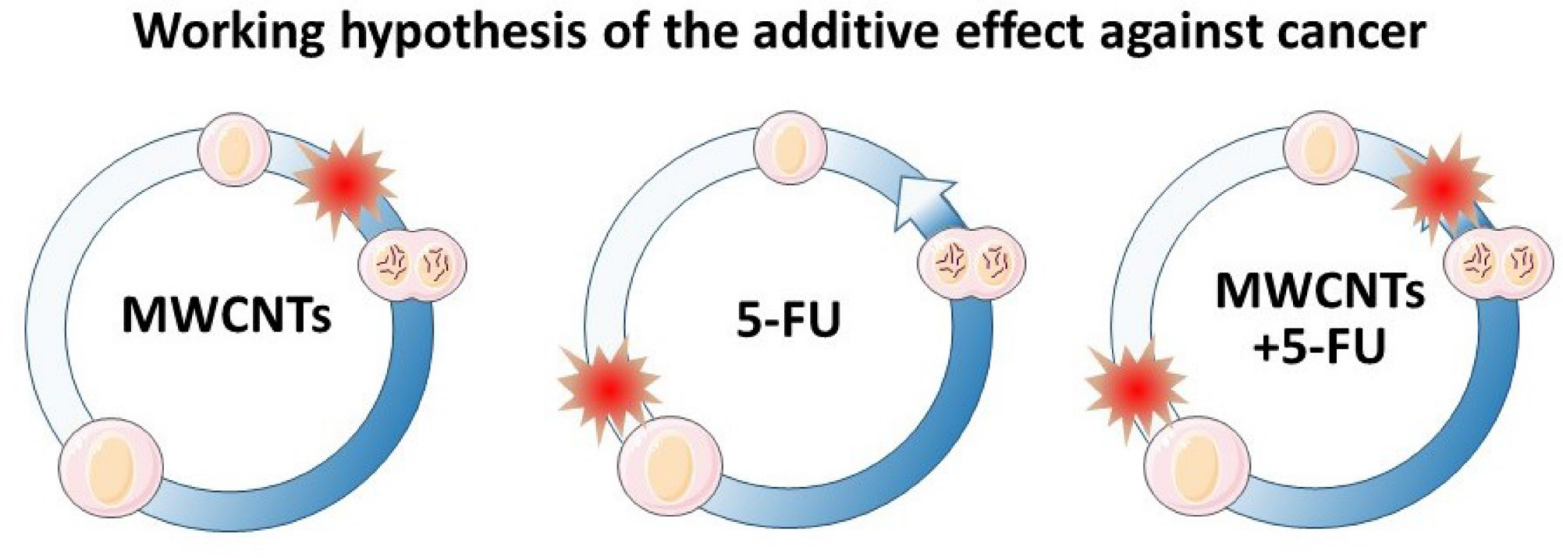
Cell cycle MWCNTs and 5-FU blockage points (**left**) MWCNTs typically interfere with the mitosis process. (**center**) 5-FU blocks the cell cycle at the “S” phase, during DNA and organelle replication. (**right**) Sequential and complementary blockage points of MWCNTs and 5-FU therapies. The expected double blockage of the cell cycle should inhibit cancel cell growth more intensively.

But despite these numerous advantages, the stigma of the structural similarity of CNTs with asbestos fibers has slowed down progress of these nanomaterials in medicine, and have been repeatedly excluded in the design of drug nanocarriers [20]. Fortunately, the recent discovery of a series of surface treatments that make MWCNTs more biocompatible and bio-degradable by phagocytic cells, has opened many new opportunities in nanomedicine [21–27]. It is now known that macrophages *in vitro* can degrade surface-oxidized MWCNTs in few days, reducing the length of the nanotubes in approximately 30%, and *in vivo*, in the tumoral tissue after triggering significant antitumoral effects [19]. Here we investigate the possibility of enhancing the effect for traditional cancer drugs using MWCNTs as nanocarrier adjuvant systems. For the study we have loaded MWCNTs with 5-FU, a routine broadly used anticancer drug that inhibits cell replication mostly in the “S” phase of the cell cycle, complementing the intrinsic microtubule dynamics inhibitory effect of MWCNTs during mitosis (Figure 1).

## RESULTS AND DISCUSSION

### Characterization of the 5-FU physisorption on MWCNTs and *in vitro* drug release

**Figure 2:**
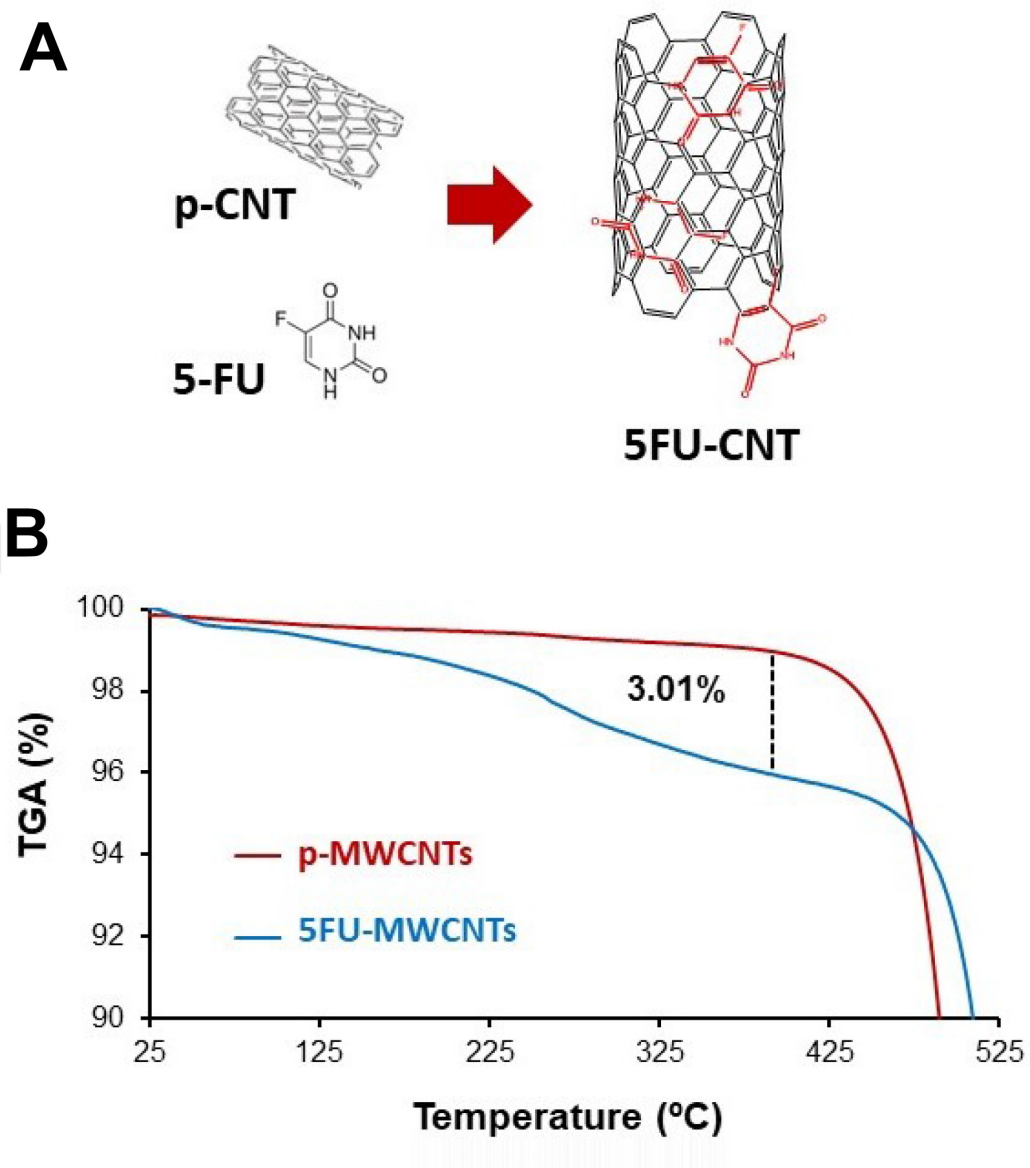
Characterization of the 5-FU-MWCNTs (**A**) Representative diagram of the interaction between CNTs and 5-FU. (**B**) TGA corresponding to p-MWCNTs and 5-FU-MWCNTs samples under air atmosphere. An approximate 3% mass loss corresponding to the physisorbed 5-FU is calculated.

Simple molecular physisorption *via* π-stacking has been broadly used for successful loading of different drugs onto graphene and MWCNTs preventing drug inactivation due to the binding procedure (Figure 2A) [28, 29]. In this study, we have physisorbed 5-FU on MWCNTs (5-FU-MWCNTs) as described in the methods section. The amount of the physisorbed 5-FU was estimated using thermogravimetric analysis (TGA). Figure 2B shows the TGA analysis where approximately 3% of the total mass of the 5-FU-MWCNT sample corresponds to the drug. This data was further corroborated using fluorescence spectroscopy analysis as a complementary technique (Supplementary Figures 1–2). This analysis served to verify the calculated amount of ca. 3 mg of 5-FU per 100 mg of MWCNTs (3% w/w). Hence, this data will be considered in the comparative studies that follow.

5-FU release from the 5-FU-MWCNTs was first evaluated *in vitro*. For this purpose, 5-FU-MWCNTs were exposed to physiological conditions (phosphate buffer pH = 7.4 at 37° C) during 140 h. Measurement of the collected samples using fluorescence spectroscopy suggested an initial phase of ‘burst’ release of the drug during the first 24 h, that corresponded to a 40–42% of the loaded 5-FU. This was followed by a ‘sustained release’ phase where the rest of the drug was slowly discharged in the course of several days (Supplementary Figure 3).

### Boosted anti-proliferative and cytotoxic effects to 5-FU when administered on MWCNTs

To evaluate the effect of the 5-FU loaded MWCNTs *in vitro*, we exposed HeLa cell cultures to: 3 μg/mL of the plain drug; 100 μg/mL of pristine MWCNTs (p-MWCNTs); and finally, to 100 μg/mL of 5-FU-MWCNTs. The effects of the treatments were compared 72 h after exposure to the three therapies.

**Figure 3:**
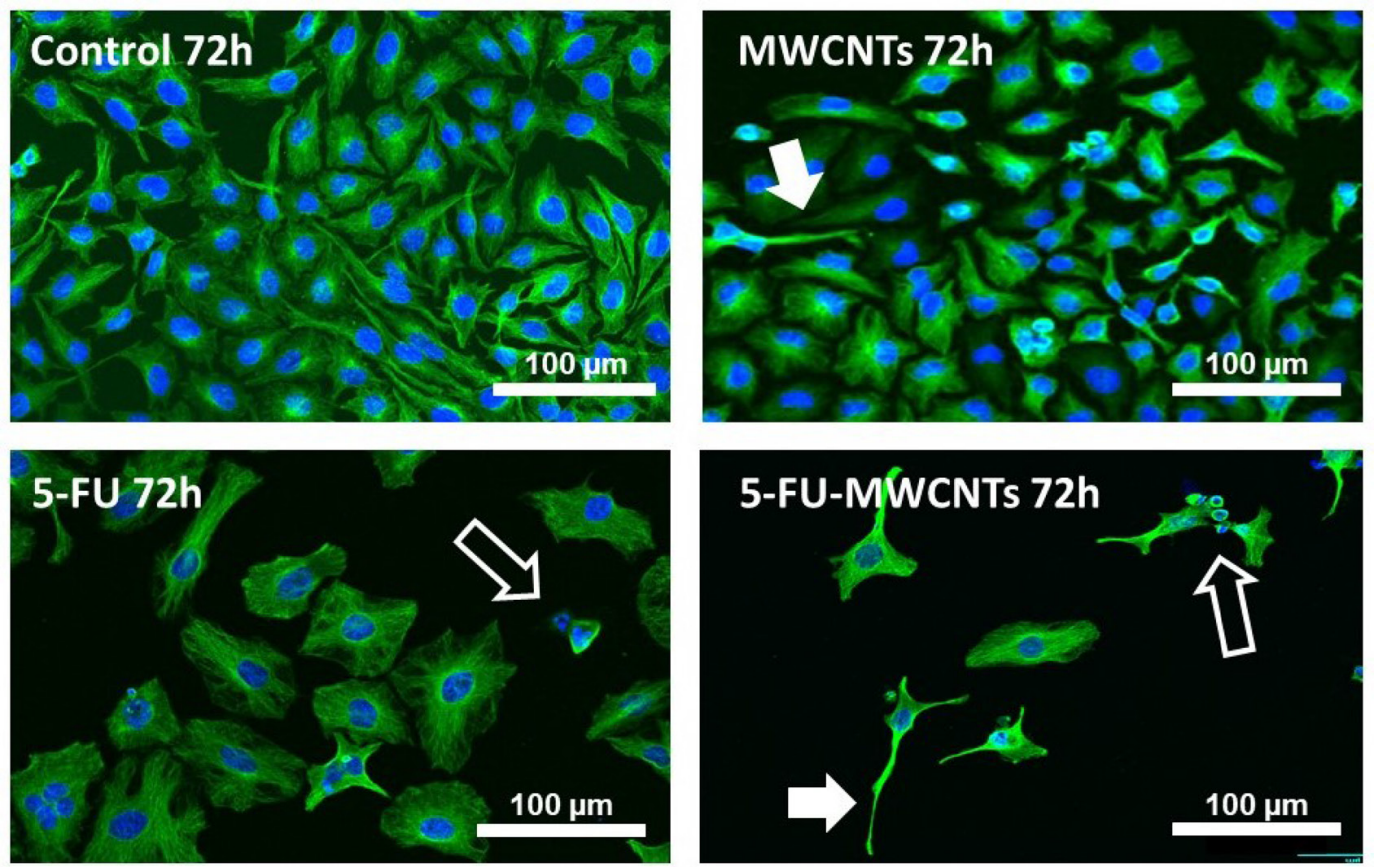
Anti-proliferative and cytotoxic effect of 5-FU-MWCNTs *in vitro* Confocal microscopy images of HeLa displaying the microtubule network (green channel) and nuclei stained with Hoechst (blue channel). Cells were either untreated (control) or exposed to 100 μg/mL p-MWCNTs, 3 μg/mL 5-FU or 100 μg/mL 5-FU-MWCNTs. Treatment with the plain drug triggered a patent increase in the cell size and patent anti-proliferative effects in the cultures. Cells treated with identical amounts of 5-FU loaded on MWCNTs displayed an enlarged size and apparent magnification of 5-FU anti-proliferative effects. As typical for MWCNTs, some cells also presented long cellular extensions resulting of bundled microtubules (solid arrows). Cells dying by apoptosis are also observed (empty arrows).

Both, the resuspended 5-FU and 5-FU-MWCNTs produced a potent anti-proliferative effect accompanied by an obvious increase in the cellular size compared to untreated controls, or to cells treated with p-MWCNTs. This phenotype suggested a possible blockage in the “S” part of the cell cycle (Figure 3). Remarkably, cultures treated with 5-FU-MWCNTs appeared more severely affected that those treated with the free drug or p-MWCNTs.

Quantification of the cellular area in cultures treated with both, 5-FU and 5-FU-MWCNT, revealed a statistically significant 3-fold increase in cell size compared to untreated or p-MWCNTs controls (Supplementary Figure 4). As previously reported for MWCNTs [9], cells treated with 5-FU loaded onto MWCNTs also displayed characteristic long and thin cytoplasmic extensions resulting of the biomimetic interaction between MWCNTs with the cytoskeletal microtubules (Figure 3, solid arrows, Supplementary Figure 4). In addition, these cultures also presented the expected low surviving cellular rate. The abundance of cells with patent signs of apoptosis (empty arrows) supported the efficiency of this cytotoxic treatment *in vitro* and suggested a significant rise in the efficacy of the drug when applied decorating MWCNTs.

### A double cell cycle blockage to intensify the therapeutic effect

To perform an accurate qualitative and quantitative analysis of the cytotoxic effects of the treatment, we used flow cytometry. This statistically powerful technique allows the characterization of the cell cycle for each condition, and to quantify the percentage of apoptotic cells in approximately 10,000 cells per experiment. For the study we used human HeLa cells and murine B16F10 cells. This murine model of malignant melanoma cells is resistant to many traditional chemotherapeutic drugs [30] and, as the majority of melanomas, displays an aggressive nature, being genetically heterogeneous and highly metastatic [31].

**Figure 4:**
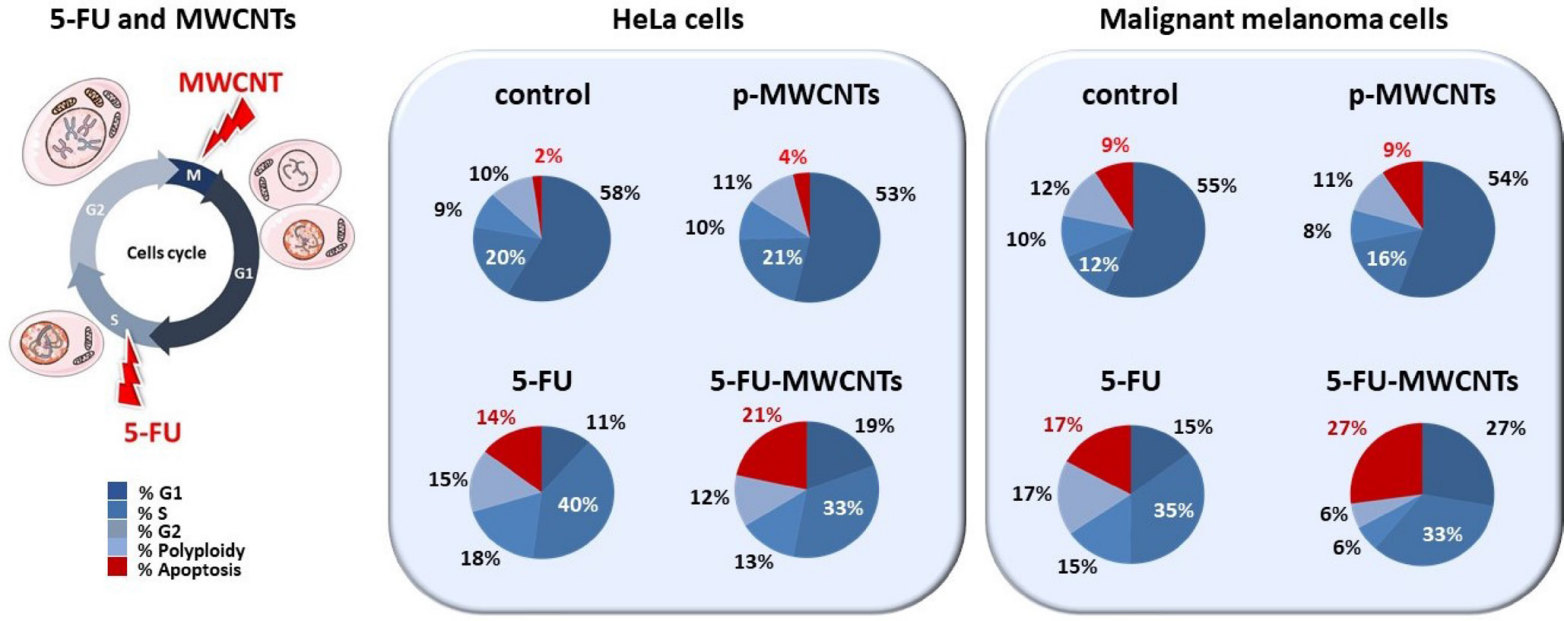
Quantification of the cytotoxic effect of 5-FU-MWCNTs compared to controls Cultures of HeLa and malignant melanoma cells were exposed to p-MWCNTs, soluble 5-FU or 5-FU-MWCNTs during 72 h and were compared to untreated controls. Flow cytometry quantitative analysis demonstrates that the cytotoxic effect of the 5-FU when administered as 5-FU-MWCNT is significantly enhanced in both, human and murine cancer cells (represented in red). Cell cycle changes evidence a patent blockage in the “S” phase for both, 5-FU and 5-FU-MWCNTs treatments (indicated with white lettering).

Figure 4 illustrates the proportion of live cells (blue) vs. dead cells (red) for untreated controls and for the 3 different treatments: p-MWCNTs, 5-FU, and 5-FU-MWCNTs. These data reveal a significant blockage in the “S” phase, 72 h post-treatment typical of 5-FU for both, HeLa and melanoma cells. More interestingly, both cell lines showed higher sensitivity to the cytotoxic effect of the 5-FU-MWCNTs compared to the plain drug. Cytotoxicity in cells treated with standard 5-FU was 14% and 17% for HeLa and melanoma cells, respectively. Parallel cultures treated with the same amount of 5-FU physisorbed onto MWCNTs displayed 21% and 27% cell death, respectively (Figure 4, red). Furthermore, the sum of the cytotoxic effects of 5-FU and the p-MWCNTs separately were less than the effect of the 5-FU-MWCNTs (4%+14% <21% for HeLa, 9%+17% <27% for the melanoma cells). These results suggest that, at least *in vitro*, MWCNTs increase the effectiveness of the drug while significantly amplifying the cytotoxic effect of the two chemotherapies individually applied. Therefore, data indicate that the sequential inhibitory effect of the 5-FU at the “S” phase and the MWCNTs at the “M” phase of the cell cycle, as depicted in Figure 1, could be complementary.

### A significantly enhanced *in vivo* effect of 5-FU-MWCNTs compared to 5-FU

**Figure 5:**
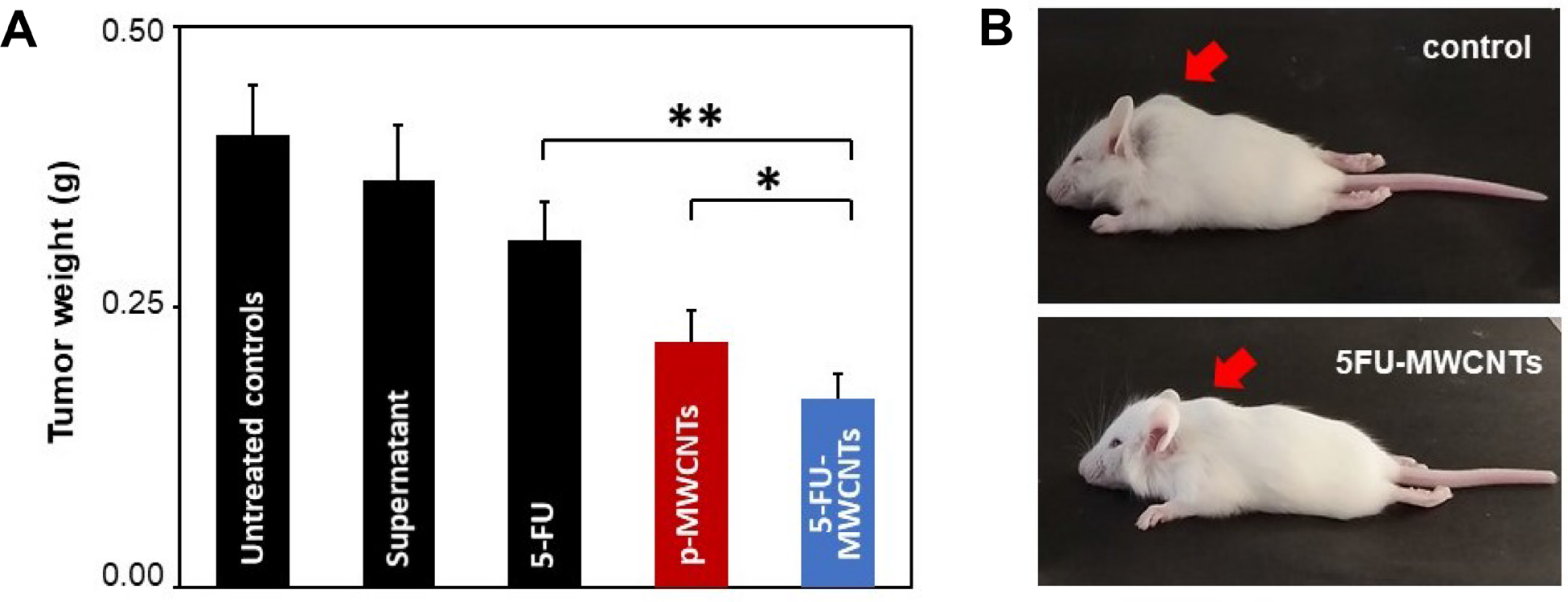
*In vivo* 5-FU-MWCNTs effects in solid melanoma tumours (**A**) Statistical evaluation of the average tumoral weight 96 h post-treatment (single injection containing 2 μg of p-MWCNTs, 5-FU-MWCNTs or identical amounts of the 5-FU drug). 5-FU-MWCNTs trigger a statistically significant anti-tumoral effect respect to 5-FU injected locally (*t* = 3.6, *n* = 75, ^**^= *t*_*.99*_) or tumours treated with plain p-MWCNTs (*t* = 1.31, *n* = 87, ^*^= *t*_*.975*_). (**B**) Representative mouse littermates bearing solid melanoma tumours 96 h after a single intra-tumoral injection of a control resuspension medium or 5-FU-MWCNTs. Arrows point at the tumour location.

The anti-tumoral effect of 5-FU-MWCNTs was also tested on solid melanoma tumours produced by transplantation of B16-F10 cells. This system model is highly representative of most malignant tumours, being characterized by local acidosis, edema and abundant tumor-associated supporting stromal cells that include macrophages [32]. As in previous studies, solid pigmented melanoma tumours were treated only once to improve reproducibility, with either 5-FU-MWCNTs (2 μg) or the controls, namely: (i) the control resuspension media (Figure 5, supernatant), (ii) resuspended p-MWCNTs (2 μg), or (iii) the free drug (identical amount as those supported on the 2 μg of MWCNTs). For direct comparison, injections were performed in littermates, in a total population of more than 200 mice. All mice were sacrificed 4 days post-treatment for analysis.

Results shown in Figure 5 demonstrate how 4 days after a single treatment with 5-FU-MWCNTs there was an important reduction of the tumor mass. Tumors treated intratumorally with the plain drug or p-MWNCTs were reduced compared to controls but were significantly larger than those treated with de 5-FU physisorbed onto MWCNTs. Compared to the control treatment, tumoral masses were almost half the size when treated with 5-FU-MWCNTs. This effect was significantly improved respect to that triggered by the plain drug or plain p-MWCNTs. The later also demonstrated a patent tumoral growth inhibitory effect confirming previously reported data [17]. Summarizing, these experiments served to conclude that the important antitumoral effect observed for the 5-FU loaded onto MWCNTs -reducing in ca. 60% the tumoral mass- results of the complementary cytotoxic mechanisms generated by the two therapies, interfering with two different steps of the cancer cell proliferative cycle, as originally hypothesized in Figure 1.

## MATERIALS AND METHODS

### Materials

High-purity (>98%) MWCNTs were obtained from Sigma Aldrich (Ref. 698849). The *as-produced* MWCNTs were washed with HCl to remove impurities, resuspended in distilled water by sonication and incubated with a solution of ca. 10 mg/mL 5-FU (Accord, Ref. 603544.3). The mixture was stirred in a vertical wheel at ca. 20 r.p.m. for 2 h at room temperature, centrifuged at 12000 g and washed with distilled water three times in repeated cycles of centrifugation (12000 g, 5 min)/redispersion (Supplementary Figure 1).

The concentration of the physisorbed 5-FU was estimated using fluorescence spectroscopy as described in the Supplementary Material (Supplementary Figures 1–2). TGA was carried out in a TG-DSC Setaram Model Setsys Evolution 1750 in air atmosphere. Data were processed with the Quadstartm 422 software (T ramp 10.00° C/min to 830.00° C). 5-FU-Release assays were performed in PBS (pH = 7.4) at 37° C. Released 5-FU was measured using fluorescence spectroscopy in an Edinburgh Inst. FLSP-920 using the calibration line of Supplementary Figure 2.

### Cell culture and confocal microscopy imaging

HeLa cells were grown in cultured with Eagle’s Minimum Essential Medium (Biowhittaker™). Cells were fixed with 4% paraformaldehyde for imaging. DNA was stained with Hoechst dye (from Sigma-Aldrich^®^). Microtubules were immunostained with B512 anti- α-tubulin antibody (Sigma-Aldrich^®^) and a secondary goat anti-mouse Alexa Fluor 488-conjugated IgG (Molecular probes^®^). Confocal laser scanning and phase contrast images were obtained with a Nikon A1R confocal microscope. Image processing was performed with the NIS-Elements Advanced Research software. All fluorescent images are pseudo-colored.

### Tumor studies *in vivo*

Tumorigenesis was induced by subcutaneous transplantation of a total of 2 × 10^5^ B16-F10 murine melanoma cells in 10 µL of culture medium containing antibiotics following previously described protocols [18, 19, 32]. Animal experimentation procedures were performed according to EU legislation in accordance with the Guidelines for ‘Care and Use of Laboratory Animals’ of The University of Cantabria and were approved by the local Animal Ethics Committee. Solid pigmented tumours were single treated 7 days post-transplant with a unique intratumoral dose of 2 µg of nanotubes resuspended in a volume of 10 µL of culture medium. Parallel experiments comparing the effect of 5-FU-MWCNTs, pristine MWCNTs (p-MWCNTs), 5-FU (using equivalent calculated amounts) or resuspension media -as an excipient control- were performed. To reduce natural artefacts, litters were divided in two halves and were injected in parallel with two of the former compositions. Tumours masses were carefully dissected and weighed 4 days post injection for quantitative statistical analyses. Total number of animals are indicated in the text and figure.

### Flow cytometry and electron microscopy imaging

Flow cytometry studies were carried out on a suspension of fixed cells stained with Hoechst using a Becton Dickinson FACS CantoII equipment. This dye produces a quantitative staining of DNA that allows the determination of changes in the cell cycle and cell proliferation blockage, permitting DNA fractional quantification (“sub-G1/G0” peak) indicative of apoptosis. Three different replicas of the experimental analysis were performed on an average of 10,000 cells per condition using the FACS Diva software (Becton Dickinson). For illustrative purposes the original flow cytometry graphs have been substituted by pie charts displaying the calculated proportions of the different cell populations, including apoptotic cells in Figure 4. Transmission Electron Microscopy (TEM) was performed in a JEOL JEM 2100 operated at 120 kV on ethanol-dispersed samples adsorbed onto a Lacey copper grid.

### Statistical analyses

A Student’s two-tailed *t-*test was used for statistical analysis and to evaluate significance that was stablished for a (^*^) *p =* 0.05 or a (^**^) *p =* 0.01. The confidence levels and total number of events included in the study (*n*) are indicated in the figure legends. Quantitative results are expressed as mean values with their corresponding standard error bars.

## CONCLUSIONS

The emergence of drug resistance depends on the genetic instability, heterogeneity and high mutational rate of tumour cells among others. Malignant cancer cells continuously develop new mechanisms of resistance to chemotherapy that include drug destruction, selection of mutations that inhibit drug binding to targets, drug efflux out of the cells, etc. The administration of chemotherapy loaded onto MWCNT used as nanocarriers can significantly improve many different aspects of the traditional chemotherapy. On one hand, the penetrating properties of these nanomaterials are interesting when considering, for instances, treatments applied topically, where CNTs can penetrate and spread into the affected lesion transporting the drug away from the application point. But more interestingly, MWCNTs have intrinsic antitumoral properties that can be exploited in cancer treatment interfering with microtubule dynamics, triggering effects similar to traditional drugs such as Taxol^®^ (paclitaxel) or Epothylones. Indeed, the fact that MWCNTs can produce notable antitumoral effects in solid tumours generated by Taxol^®^-resistant cells suggests these nanofilaments can complement and significantly boost chemotherapy. Here we demonstrate how drug and nanomaterial therapies can complement each other, *in vitro* and *in vivo* in the treatment of cancer. In conclusion, these data invite to improve anti-cancer delivery systems considering the use of nanomaterials with intrinsic antitumoral properties as active excipients, to enhance the therapeutic effect of traditional chemotherapy, preventing drug resistance in cancer.

## SUPPLEMENTARY MATERIALS FIGURES



## Abbreviations

CNTs: Carbon nanotubes
MWCNTs: multi-walled nanotubes
5-FU: 5-Fluorouracil
TGA: thermogravimetric analysis
TEM: Transmission electron microscopy
