# Supplementary material for: Multi-walled carbon nanotubes complement the anti-tumoral effect of 5-Fluorouracil
Source: Oncotarget. 2019 Mar 12;10(21):2022–9. doi: 10.18632/oncotarget.26770 (PMC6459348; doi:10.18632/oncotarget.26770)
Supplement: Supplementary file 1 [file oncotarget-10-2022-s001.pdf]

# Multi-walled carbon nanotubes complement the anti-tumoral effect of 5-Fluorouracil

## SUPPLEMENTARY MATERIALS

## APPENDIX A

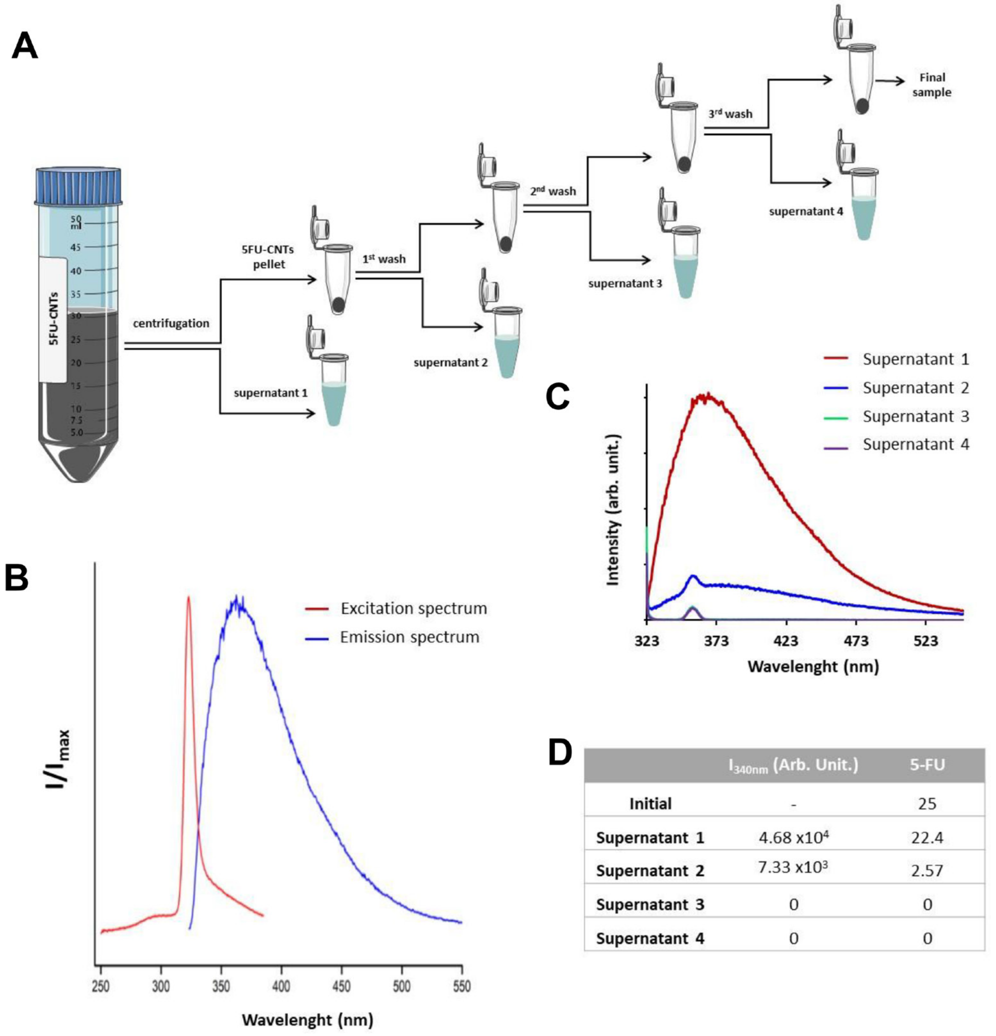


| | $I_{340nm}$ (Arb. Unit.) | 5-FU |
|---|---|---|
| Initial | - | 25 |
| Supernatant 1 | 4.68 x10$^{4}$ | 22.4 |
| Supernatant 2 | 7.33 x10$^{3}$ | 2.57 |
| Supernatant 3 | 0 | 0 |
| Supernatant 4 | 0 | 0 |

**Supplementary Figure 1: 5FU-MWCNTs sample characterization.** (**A**) Representative scheme of the process carried out to wash 5-FU-MWCNTs sample. (**B**) Excitation spectra (red) and emission (blue) of 5-FU. The spectra are normalized to the maximum intensity. The excitation spectrum was carried out by monitoring intensity at $\lambda = 455$ nm. The emission spectrum was obtained by exciting at $\lambda = 317$ nm, giving a maximum emission at $\lambda = 363$ nm. (**C**) Fluorescence emission spectra of supernatant (red line) and the sequential washes (blue, green and purple lines). As we start with a known concentration of 5-FU, we have subtracted the correspond concentrations from the supernatant and washes, thus, we obtain a 3% of the mass of the sample corresponds to 5-FU. (**D**) Calculated concentration of residual 5-FU in the supernatants after repeated washing. The intensities were taken at 340 nm wavelength.

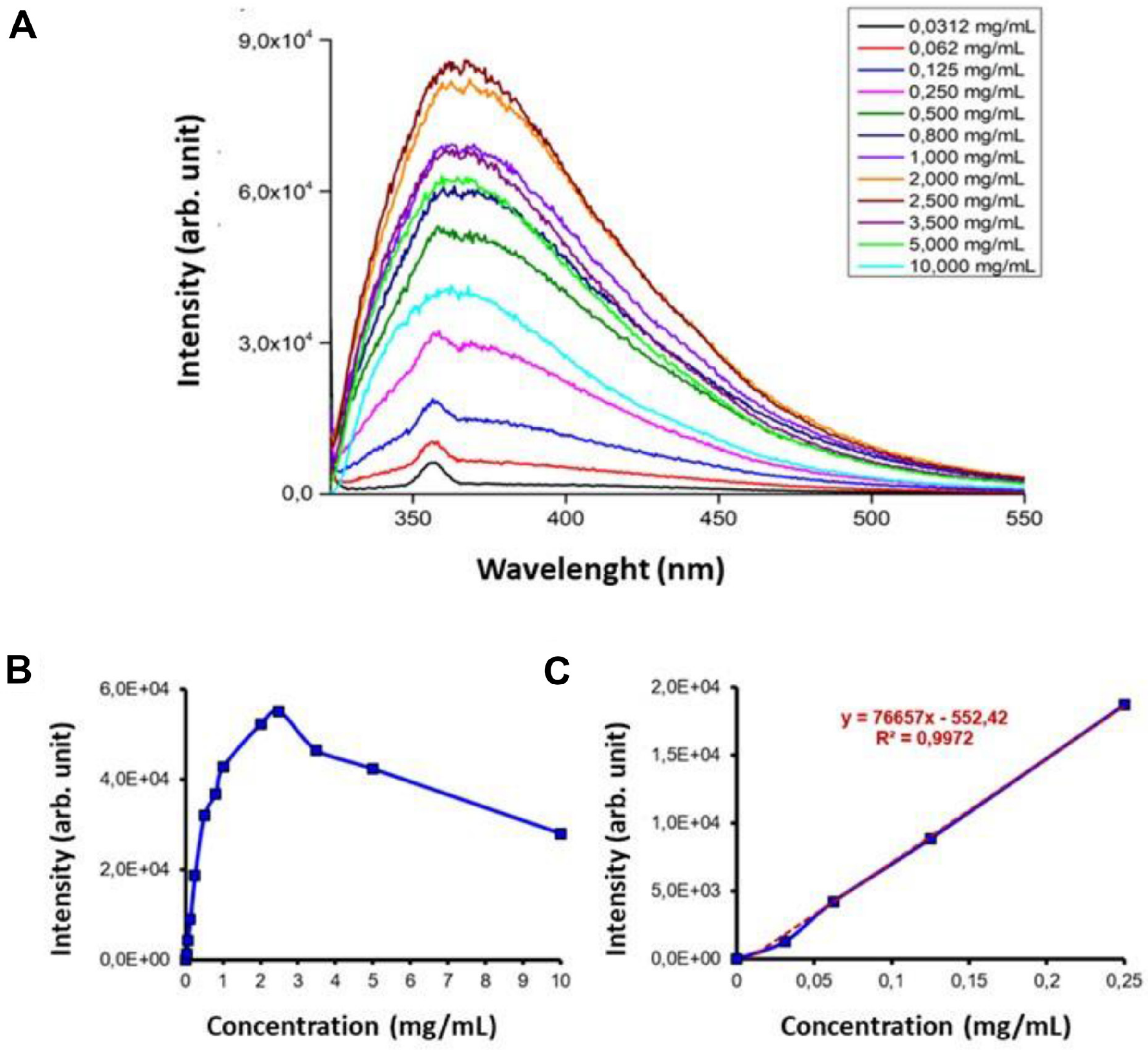


**Supplementary Figure 2: Calibration curve of 5-FU using fluorescence spectroscopy.** (**A**) 5-FU emission spectra for a range of concentrations between 0.0312 and 10 mg/mL. At low concentrations (between 0.25 and 0.0312 mg/mL) a band begins to appear (maximum intensity at 356 nm) that corresponds to the signal of water. (**B**) Calibration curve of 5-FU for fluorescence intensities corresponding to ʎ = 340 nm. From 2.5 mg/mL the emission intensity begins to decrease, so for the same emission intensity there will be two possible concentrations. (**C**) At low concentrations the relationship between emission intensity and concentration is linear with R2 = 0.9972.

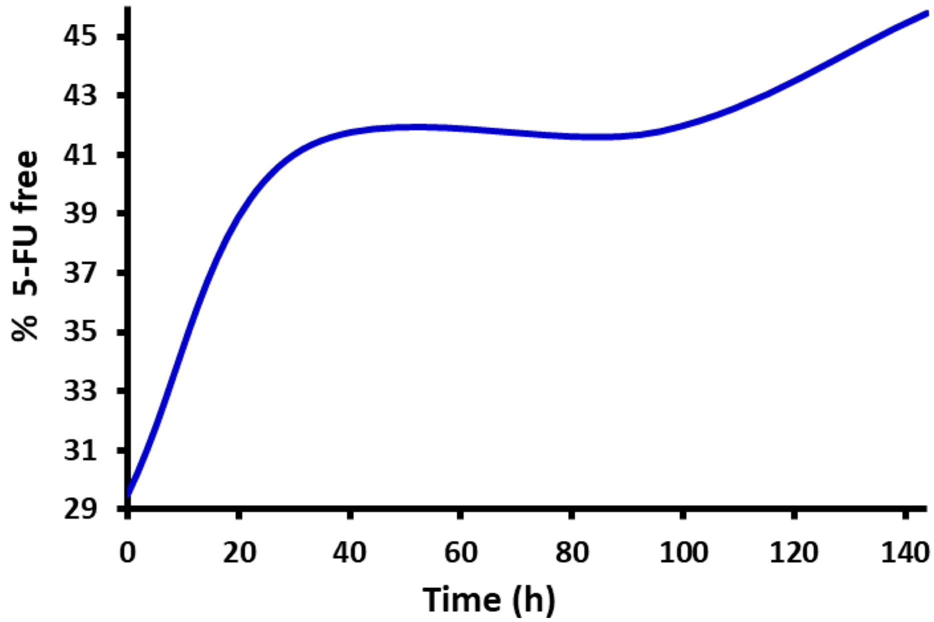


**Supplementary Figure 3: In vitro 5-FU release assay.** 5-FU release of 5-FU-MWCNTs upon exposure to physiological conditions (PBS 1× at 37° C).

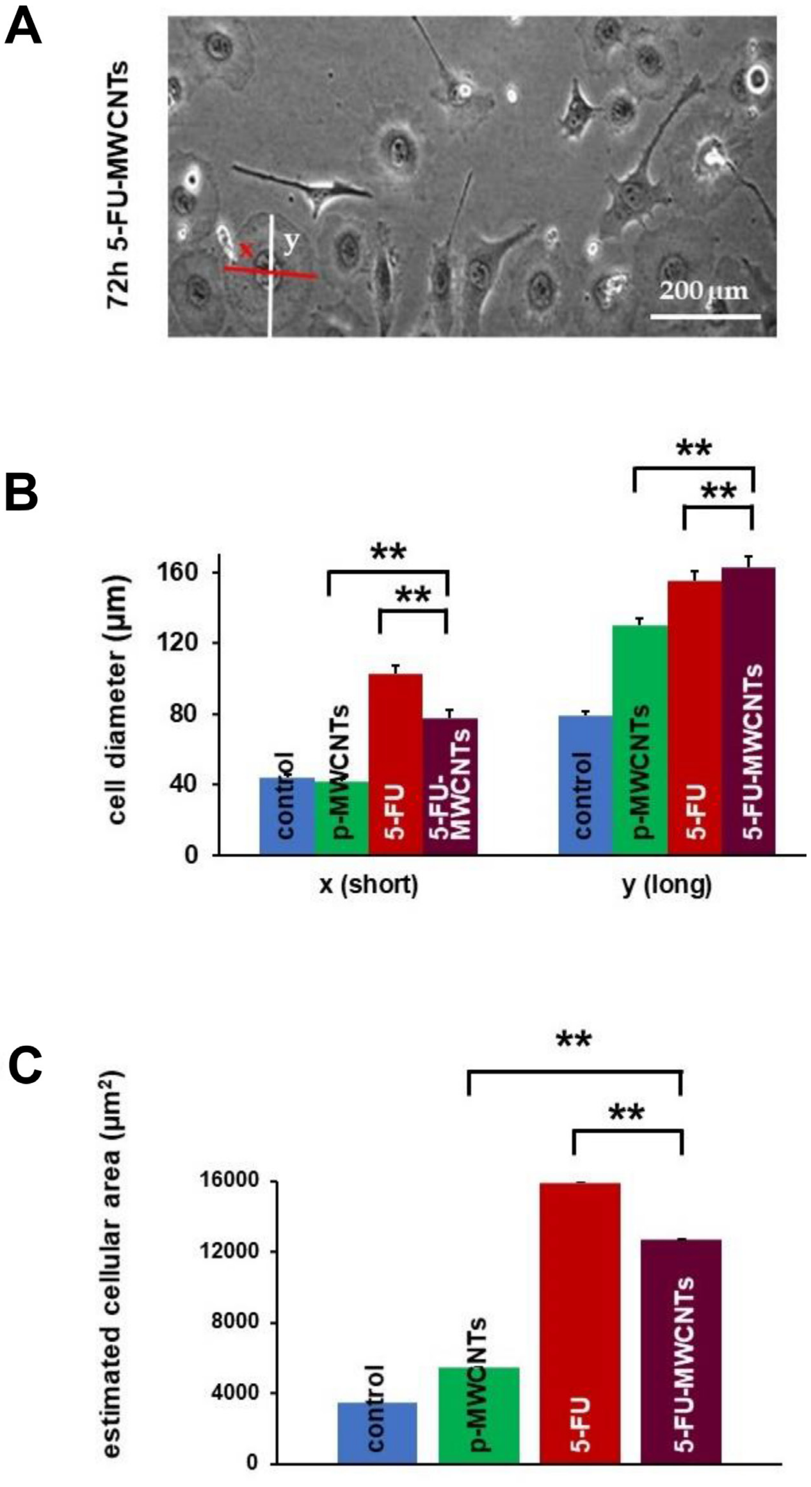


**Supplementary Figure 4: Statistical analysis of the changes in cell size.** (**A**) Representative phase contrast image of the cells treated with 5-FU-MWNCTs, where representative measurements along the X (red) and Y (white) axes are indicated. (**B**) Histograms representing the estimated cellular lengths along the X and Y axes upon cell exposure to the different treatments ($n$ = 150, $^{**}= t_{.9995}$). (**C**) Histogram of the estimated cellular area resulting of the multiplication of X and Y axes ($n$ = 150, $^{**} = t_{.9995}$). the estimated areas of the HeLa cells upon exposure to the different treatments ($n$ = 150, $^{**} = t_{.9995}$).
